# A hot-blast warming facility for simulating global warming in low-stature crop systems and its application case to assess elevated temperature effects on rice in Central China

**DOI:** 10.1186/s13007-020-00598-1

**Published:** 2020-04-23

**Authors:** Zuolin Zhang, Zhiyuan Yang, Shah Fahad, Tong Zhang, Wenhao Xu, Kehui Cui, Shaobing Peng, Jianliang Huang

**Affiliations:** 1 National Key Laboratory of Crop Genetic Improvement, MOA Key Laboratory of Crop Ecophysiology and Farming System in the Middle Reaches of the Yangtze River, College of Plant Science and Technology, Huazhong Agricultural University, Wuhan, 430070 Hubei China; 2 Hubei Collaborative Innovation Center for Grain Industry, Yangtze University, Hubei, China; 3 Department of Agriculture, University of Swabi, Khyber Pakhtunkhwa, Pakistan

**Keywords:** Climate change, Open-top chamber system (OTCs), Convection heating, Wind break, Rice (*Oryza sativa L.*), Grain yield

## Abstract

**Background:**

To study the impact of climate warming on crops, it is crucial to have a warming equipment suitable for their field environment. A facility is needed that can provide suitable combinations of different temperatures at reasonable cost for large plots.

**Results:**

Here, an additional field warming facility option named the hot-blast warming facility (HBWF), which comprised heaters, blowers, wind breaks, and a control board was developed. An application case based on HBWF was carried out to assess elevated temperature effects on rice in Central China during 2015 and 2016. We tested four elevated temperature treatments on four rice cultivars under paddy field conditions and measured yield and its components. Heating convection air directly, the facility could increase the temperature of the rice canopy up to 1–2 °C, which could properly simulate global warming. Considering the costs, the HBWF reduced the operating costs because of its relatively lower power consumption (0.164 kW/m^2^), which was 80% lower than that of Free Air Temperature Increase. Our results demonstrate that the HBWF could build a 25 m^2^ homogeneous heating area and had little effect on the relative humidity under a paddy field environment. Warming treatments significantly reduced the grain yield by 4.4–22.7% in 2015, and 30.8–61.9% in 2016, compared to the control. The main contribution to the significant decrease of the grain yields was the decrease in seed setting rate. Moreover, a reduction of 1000-grain weight led to the decline in grain yield. The increasing ranges of the temperature simulated by HBWF were stable in different years, however, whether the elevated treatments demonstrated significant difference on rice growth mainly decided by the basic atmospheric temperature (as the control) during the growth period.

**Conclusions:**

The new warming facility is suitable for field trials to assess elevated temperature combinations and provides an extra equipment option for use in elevated temperature research in the future.

## Background

Global mean surface temperature will increase 0.3–4.8 °C from 2081 to 2100, compared to that from 1986 to 2005 [1]. Under global warming, not only the days and intensity of precipitation will change [2], but also the increase of the daily maximum temperature is generally lower compared to the daily minimum temperature [3–5]. It means that the daily temperature difference range (DTR) will decrease and the warming may also have an asymmetric effect. Previous reports based on crop models [6, 7] and historical data analyses [8, 9] showed that warming during daytime and nighttime might significantly decrease crop yield. For instance, it’s predicted that rice yield will decrease by 10% if the daily minimum temperature increases by 1 °C [10]. From 1981 to 2010, extreme temperature stress caused by global warming resulted in a loss of about 6.1% of irrigated rice yield [11] and the yield per unit area of rice was reduced by 0.25 (0.01–0.56) t hm^−2^ 10 year^−1^ in China [12]. However, most previous studies are based on models or using pot experiments. Few studies were based on field experiments, so the actual crop production has not been exactly assessed yet [13]. Investigating the actual responses of crop growth to different temperature increasing scenarios and the underlying mechanism will provide more certain information about the effects of global warming on crop production and more theoretical supports for ensuring food security under irreversible global warming trends.

As one of the most important food crops globally, rice accounted for more than 25.8% of the total cereal production in 2017 [14]. It’s of great significance to investigate the practical effect of climate warming on rice production. To date, due to the limitation of many conditions, most previous studies were conducted under controlled conditions, such as in greenhouses [15–17] or open-top chambers [18–20]. These passive facilities are generally used to simulate elevated temperature to investigate crops’ responses in closed or semi-closed small spaces, not at the same scale as in field crop systems [21]. In this case, not only is the applied area is limited but also the daily temperature variation characteristics under natural conditions are changed. For the above reasons, it seems hard to simulate a natural environment to investigate the impact of future climate change on crop production. To overcome the deficiency of such facilities, some experts built the Free Air Temperature Increase (FATI) system, and it has been widely used to study plant responses to global warming at an ecosystem scale [22–24]. The FATI system mainly uses an infrared device to directly heat the foliage (not the air) in the field, which can provide good temperature control and has little change in the growth environment other than the warming [25–27]. Rehmani [26] reported that FATI needs to deploy six infrared heaters with a power rating of 1 kW each to warm a hexagonal 7 m^2^ plot. Too many heaters will not only produce high operating costs but also increase the number of cables in the field, which is not conducive for easy installation and disassembly. Moreover, the warming unit is completely open in the field, so its effect is more susceptible to the influence of climatic conditions such as rainfall and wind [24, 26].

For studying the impact of elevated temperatures on crop production, a site-specific test with several temperature treatments, combinations, and plot arrangements will provide more application value. However, it is difficult for existing heating facilities to satisfy the needs. To fill this gap and overcome the shortage of existing facilities, a hot-blast warming facility (HBWF) that could increase the temperature under field conditions by nearly 2 °C was developed based on the existing research results in our laboratory [28]. Herein, the assembly of the newly developed warming facility is provided in detail, and the advantages and disadvantages are also discussed. Moreover, a warming experiment with the HBWF on middle-season rice in the middle reaches of the Yangtze River was conducted to verify the feasibility of this equipment during 2015 and 2016. The objectives of this trial are developing the HBWF and testing its efficacy for assessing warming effects on rice in the field, and then provide a reliable research method to cope with the warming effect for future crop production.

## Results

### Assembly of the hot-blast warming facility (HBWF)

**Fig. 1:**
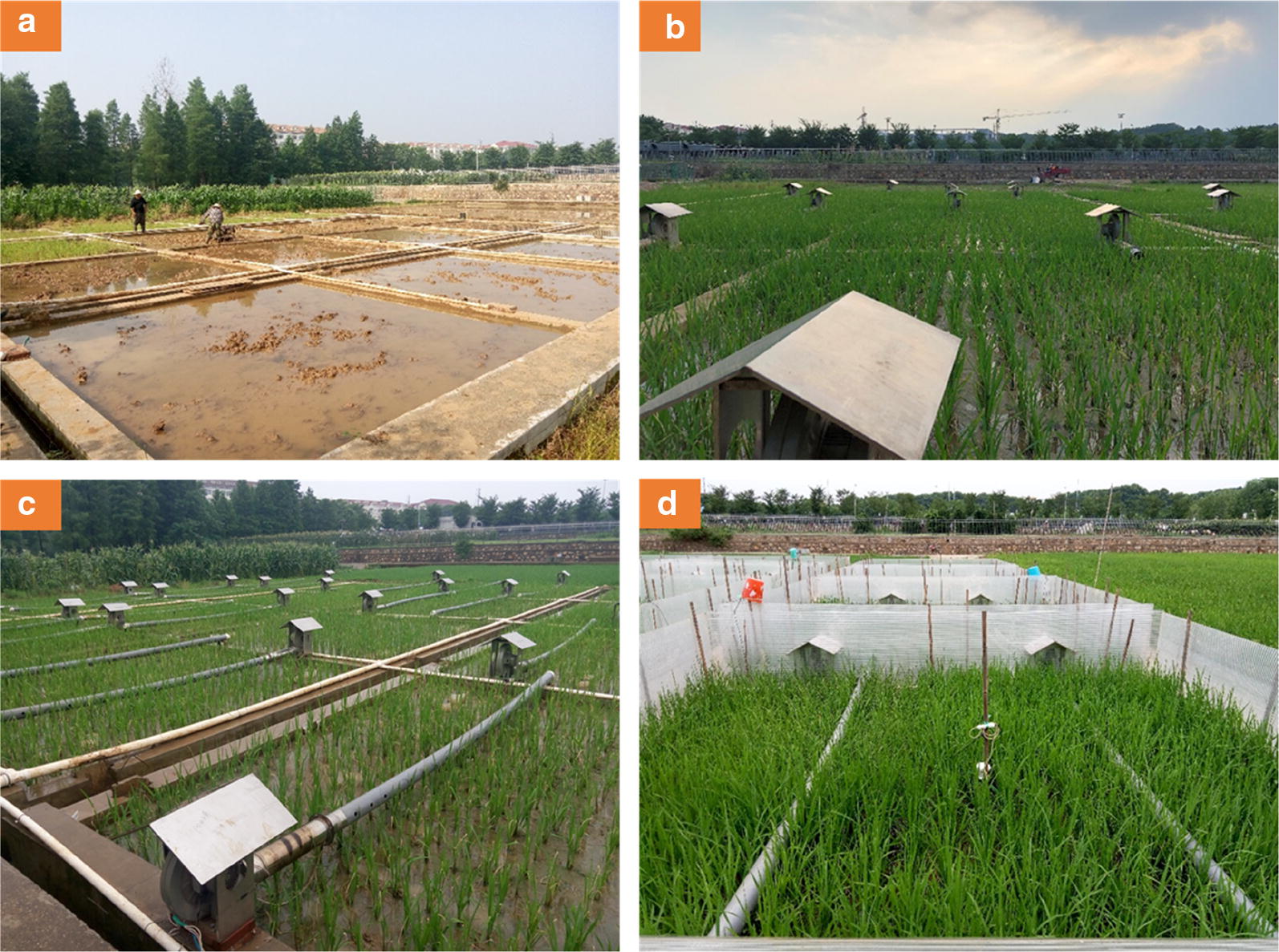
Process of the hot-blast warming facility (HBWF) installation in the field. **a** soil preparation for rice transplanting, **b**: installation of blower and heater after transplanting, **c** installing heat vent pipe, **d** installation of transparent plastic walls and temperature sensors as the last step

The HBWF installation began after transplanting rice in the paddy field. First, two blowers with heaters were fixed on a cement ridge at opposite ends of each plot by screws (Fig. 1b). Second, a pipe was connected to each blower as a heat vent to form a single heating unit (Fig. 1c). A temperature sensor (12-Bit Temp Smart Sensor S-TMB-017, sensor was sheltered from rain, sunlight and other environmental conditions by utilizing solar radiation shields to achieve more precise data about temperature and relative humidity) was installed in the center of a plot (Fig. 1d). Finally, a wind break comprised of four rigid transparent plastic walls (composed of polycarbonate, with high light transmission and less durable) (Fig. 4) was installed around the edges of the warmed plot (Fig. 1d). The farmland hot-blast warming facility increased the temperature by blowing hot air into the plot and was mainly composed of four parts: the control board, heating device, wind break and temperature measuring/monitoring unit.

#### Control board

**Fig. 2:**
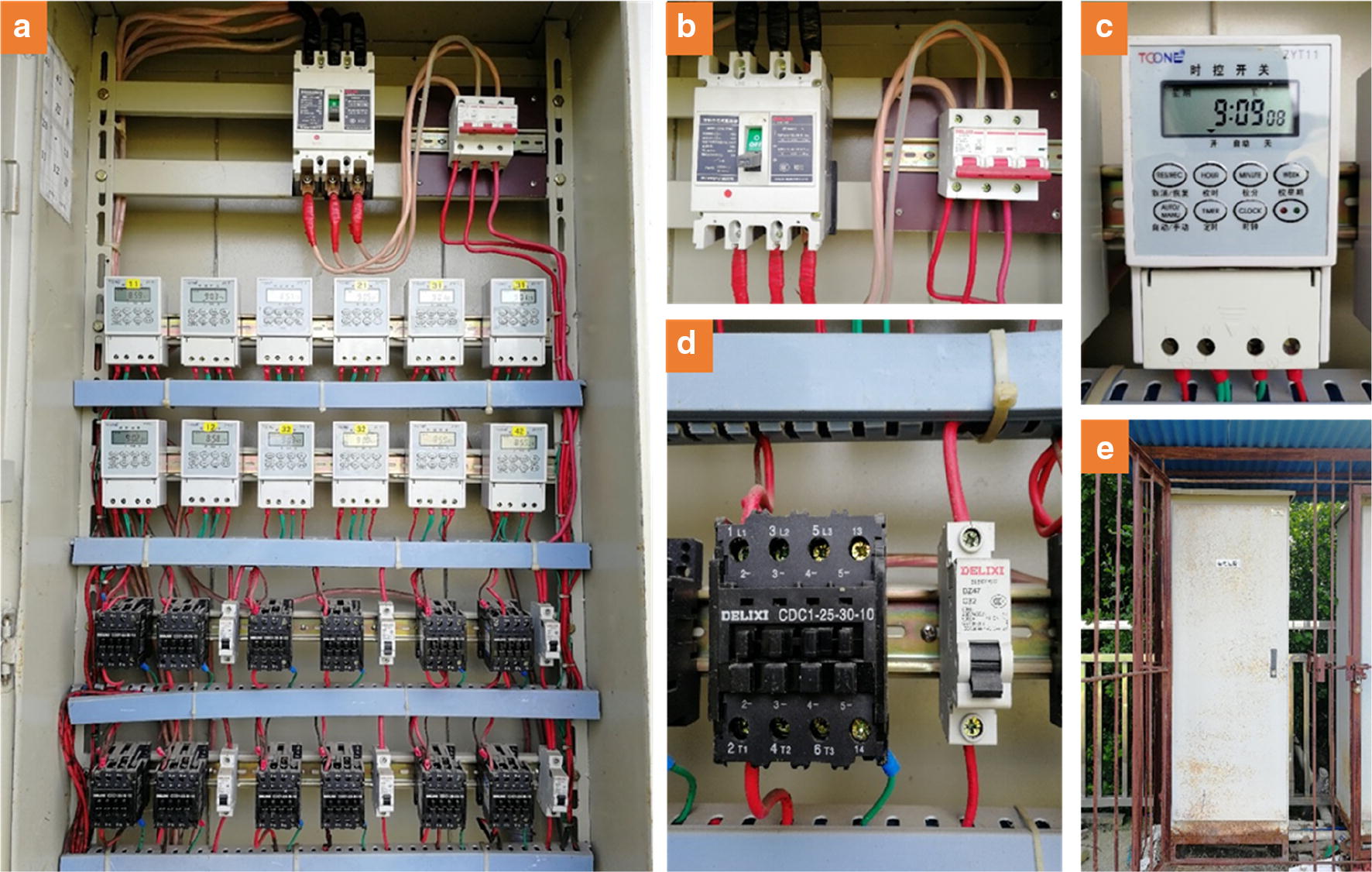
Internal structure of the electric control cabinet. **a** circuit design in the electric control cabinet, **b** main switch for power supply and main leakage protector, **c** time switch with a microcomputer, **d** relay control for each set of heating equipment and branch leakage protector, **e** external picture of the electric control cabinet

**Fig. 3:**
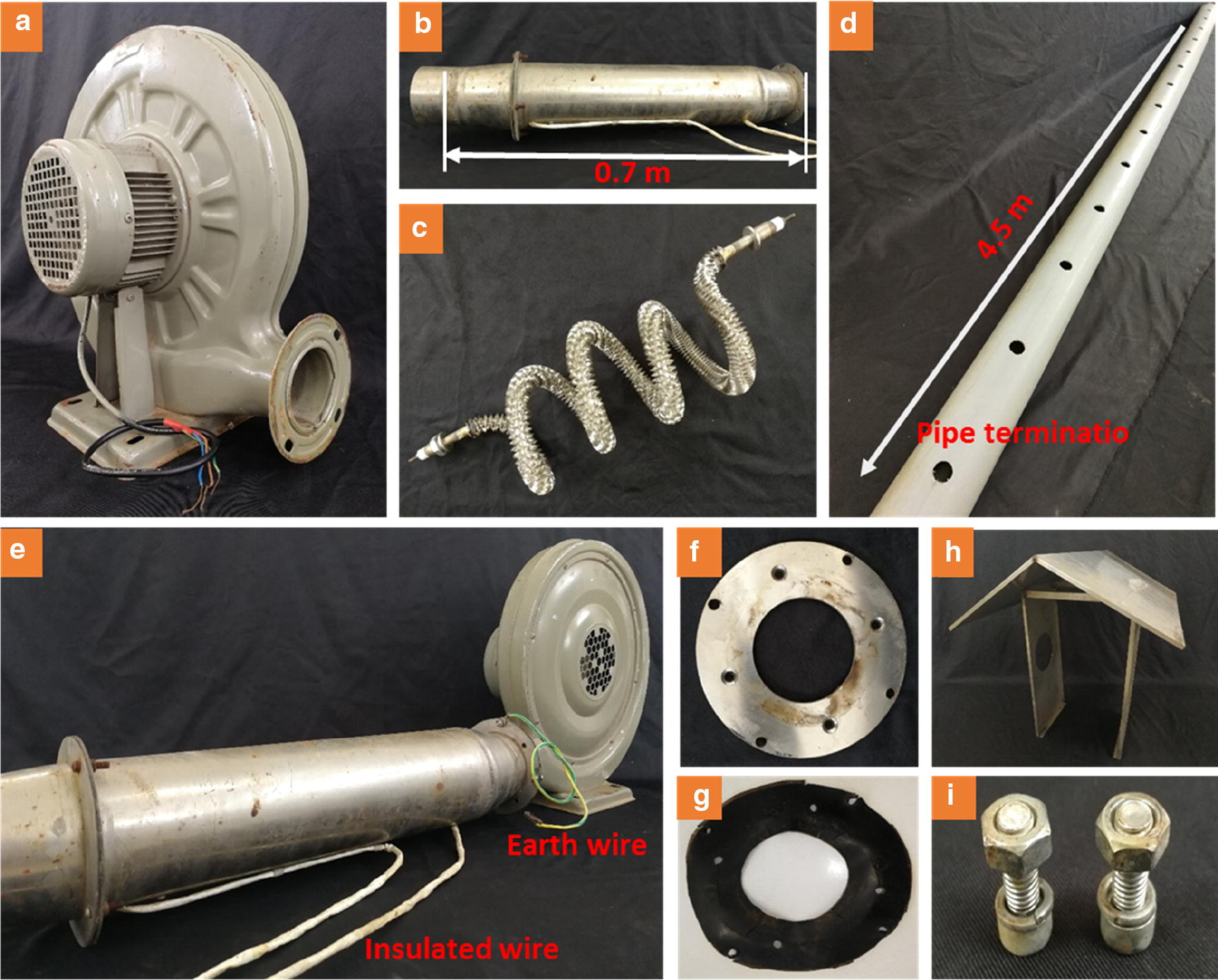
Components of heating equipment. **a** blower, **b** heater, **c** heating resistance wire, **d** heat vent pipe, **e** blower and heater, **f** steel rings, **g** rubber pad, **h** stainless steel herringbone rainproof, **i** bolt

The control board (Fig. 2a) controlled a group of heating devices by a timer with a microcomputer (ZYT11, Toone Electronic LTD, Shanghai, China) (Fig. 2c) and supplied power to the test area according to the various temperature treatments separately. A three-phase supply (380 V) was connected through the main switch to meet the electricity demand. To ensure the safety of the heating system during operation, some electrical components were installed in the electrical control cabinet with a length, width and height of 70 cm, 40 cm, and 180 cm (Fig. 2e), respectively. The installed electrical components included the main switch (CDM1-225L/3300, Delixi Electric LTD, Zhejiang, China) and circuit breaker (with functions of short circuit, overload and isolation) (DZ47-125, Delixi Electric LTD, Zhejiang, China) (Fig. 2b), a relay control (CDC1-25-30-10, Delixi Electric LTD, Zhejiang, China) for each set of heating equipment and a miniature circuit breaker (DZ47-C32, Delixi Electric LTD, Zhejiang, China) (Fig. 2d). Moreover, the electrical control cabinet and the heater in the heating device were all grounded by an earth wire. The cables from the control cabinet to the blower were all wrapped by PVC pipes (Fig. 5b) as conduits for laying, and the blower was covered with stainless steel rainproof herringbone plates (Figs. 3h, 5e) to avoid potential danger. The installation of the circuits and connecting equipment must be performed by professional electricians, so a local equipment installation company needed to employed when installing the HBWF.

#### Heating device

The heating device comprised of a blower (rated power 0.55 kW, 220 V, CZR80, Yongqiang Ventilation System LTD, Guangdong, China) (Fig. 3a), a heater (Fig. 3b) and a heat vent pipe (Figs. 3d, 5f) made up a heating device. The rotation speed of the blower is 2800 rpm, and the airflow and the wind pressure from the blower are 18 m^3^ min^−1^ and 1.7 kpa, respectively. The blower and heater were integrated through a rubber pad (Fig. 3g), bolts (Fig. 3i) and steel rings with holes (Fig. 3f), as shown in Fig. 3e. According to our technical requirements, the heater was assembled by a local company and the heat vent pipes were made by common materials in the market. The heater consisted of resistance heating wire with cooling fins (resistance of 25 Ω and rated power of 1.5 kW, 220 V) (Fig. 3c) and a stainless steel pipe projecting outward (length of 0.7 m, the diameter of 13 cm). The heating resistance wire was powered by two insulated wires (Fig. 3e) coated with high temperature-resistant rubber, and the entire heater needed to be grounded by an earth wire to ensure safety. The heat vent pipe was a high-pressure PVC-U pipe (Goody Science & Technology LTD, Hubei, China) with a thickness of 4 mm, an external diameter of 11 cm and a length of 4.5 m. The terminal of the heat vent pipe was blocked, and the pipe ran across the plot at a height of 20 cm over the ground. Twenty-one round air holes (approx. 2.5 cm in diameter) were drilled on each side of the pipe, parallel to the ground. When the wind speed is higher, the pressure is lower, so it is more difficult for thermal air to flow out when it is closer to the blower. Therefore, the distance between the holes gradually increased as follows from the blower to the end of the pipe: 16 cm, 16 cm, 17 cm, 17 cm, 18 cm,18 cm, 18 cm,19 cm, 19 cm,19 cm, 20 cm, 20 cm, 21 cm, 21 cm, 21 cm, 22 cm, 22 cm, 22 cm, 23 cm, 23 cm, 24 cm, 24 cm, 25 cm and 25 cm. This was to facilitate the uniform outflow of thermal air in a horizontal direction and increase the temperature of the plots. Also, the wind speed measured by an anemometer was nearly 0 at different heights perpendicular to the ground during the operation of the equipment. Each warming plot was equipped with two sets of heating devices, and they were positioned relative to each other with an adjacent distance of 2.5 m. In this case, the total power of each heating unit (25 m^2^) was 4.1 kW.

### Wind break and temperature measuring device

**Fig. 4:**
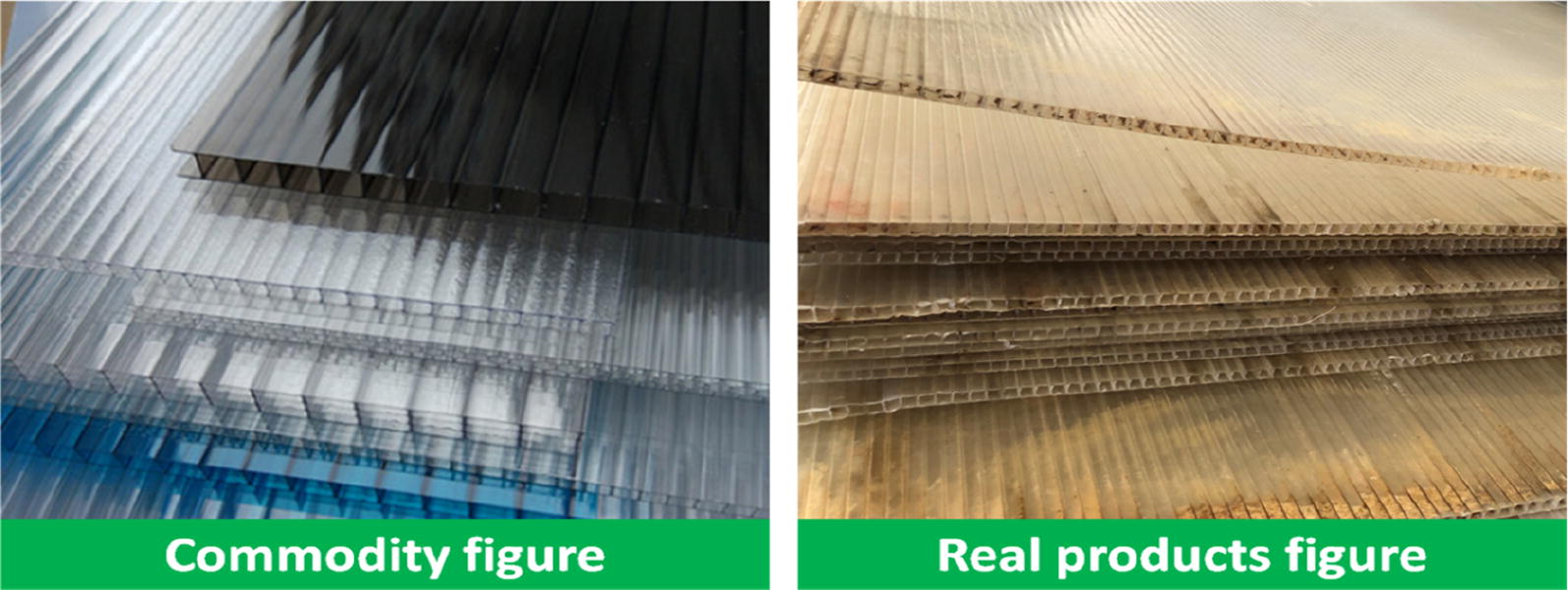
Commodity figure and real products figure about the transparent plastic walls

**Fig. 5:**
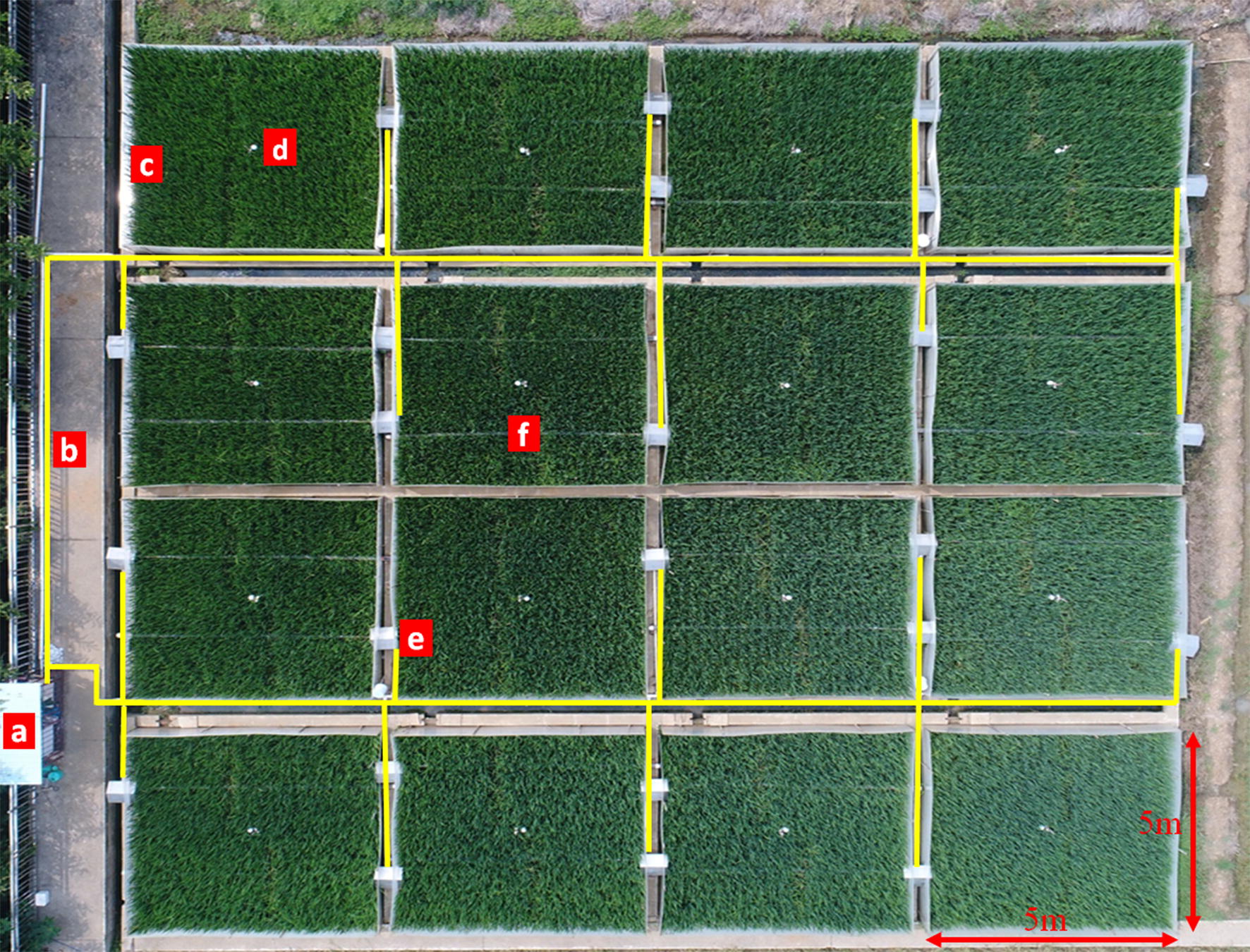
Top view of the verification test in the field. **a** electric control cabinet, **b** main electric cables laid in a PVC pipe (shown by the yellow line in the picture), **c** transparent plastic walls, **d** temperature sensors in the polt, **e** blower with herringbone rain shield, **f** heat vent pipe for warming air

To maintain constant elevated temperatures inside the plot, four high transparent plastic walls (Fig. 4) with a height of 1.1 m, a side length of 5 m and a thickness of 7 mm were used as a wind break or heat preservation device to slow the loss of heat from just blowing freely away. The wind breaks surrounded all of the various treated main plots (Fig. 5c). In effect, each plot was a large open-top chamber, albeit much wider than others described in the literature. Furthermore, each transparent plastic wall was fixed with 4–5 bamboo poles with a height of 1.2 m to prevent being blown over by the wind. Every plot formed a heating cell from the initiation of the elevated temperature treatments until the harvesting time. Ambient plots were in similar design to the heated plots except without any heating devices. During the operation of this facility, the contribution of the transparent plastic walls is limited to the warming effect by comparing the average air temperature in the control plot (29.7 °C) and the natural rice filed (29.5 °C) from 28/07 to 10/08 in 2017.

A HOBO Micro Station data logger (H21–002) and temperature sensors (12-Bit Temp Smart Sensor S-TMB-017, which could monitor temperatures from − 40 to 100 °C, Onset Computer Corporation, Massachusetts, USA) were the main components of the temperature monitoring device. The sensor was placed in the center of the main plot (Fig. 1d) and sheltered from rain, sunlight and other environmental conditions by utilizing louvered solar radiation shields to achieve more precise data. The canopy air temperature and relative humidity were recorded at 5-min intervals, and data were collected once every 30 days. All of the sensors were attached to bamboo poles, and the height was promptly adjusted to the height of the rice canopy.

### Performance of warming uniformity

**Fig. 6:**
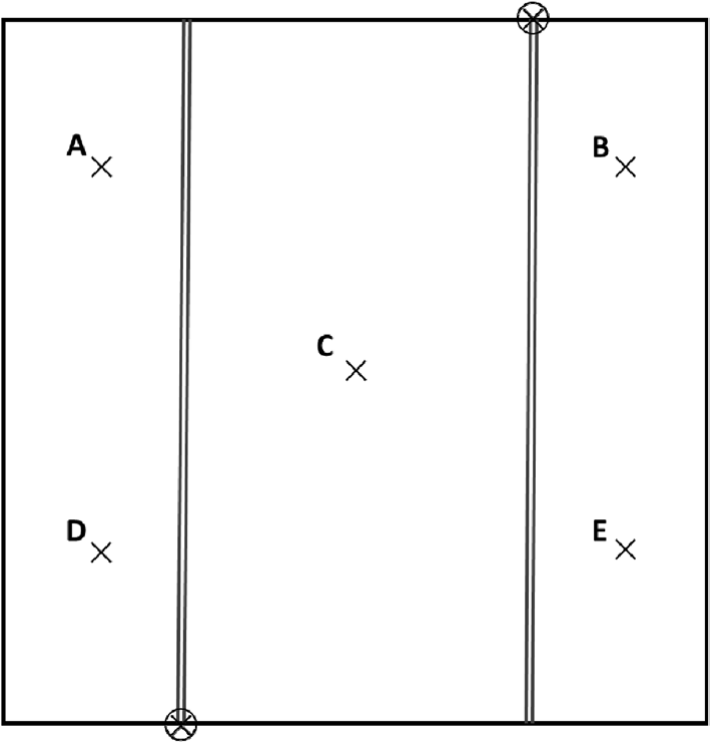
Schematic diagram to estimating spatial warming effects of a warming unit ⊗ are the blowers, × are the temperature and humidity sensors, ∥ re the PVC-U pipes, and the letters of A–E represent different temperature measuring points in a warming plot

The HOBO Microstation data logger and temperature sensors were used to evaluate the warming performance of the hot-blast warming facility after harvesting in 2016. Five temperature sensors were placed at a height of 55 cm in each plot without plants according to Fig. 6, and the heating facility was operated continuously for 5 days.

**Fig. 7:**
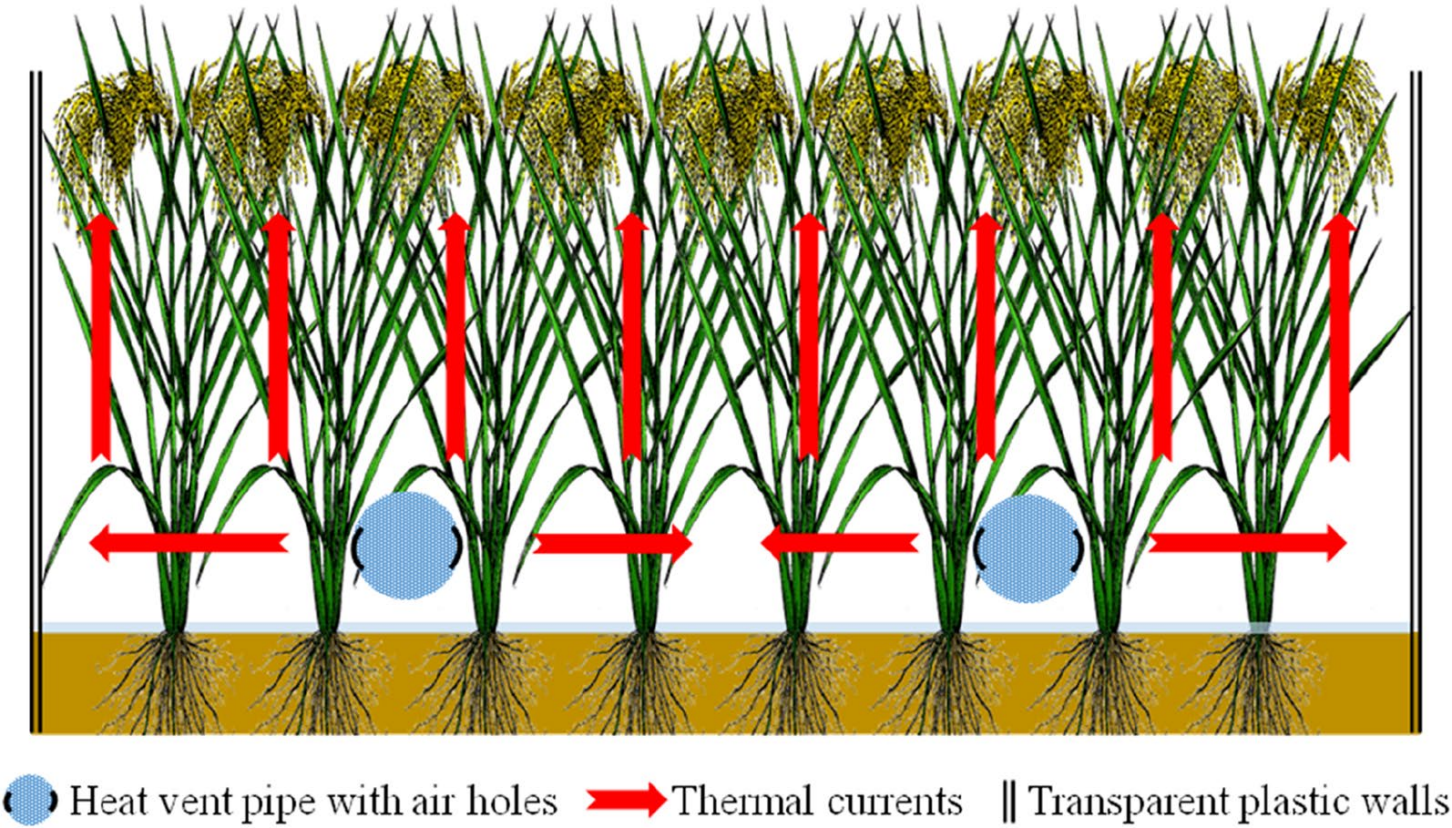
Schematic diagram of the vertical section about a warming plot

During the daytime and nighttime, the facility was able to increase the air temperature by 1–2 °C at different positions within the heating device plot unit compared with the ambient plot (Table 1). In the warming plot, the temperature increased in points A, C, and E in the daytime were approximately 1 °C, while the temperature increased in points B and D were approximately 0.6 °C, which were smaller than those of points A, C and E. In contrast, the amplitudes of nighttime temperature enhancement were significantly higher than those of the daytime, but the characteristics of the increase at each site were the same as those in the daytime. The asymmetry of elevated temperature in daytime and nighttime under the future global warming trends was simulated precisely. In the nighttime, the increases of points C and E were approximately 1.3 °C, the increases of points B and D were approximately 1 °C, and the increase of point A was relatively high, at 1.7 °C. Although the temperature increments were slightly smaller and the maximum difference of the five measuring sites was 0.5 °C in the daytime and 0.7 °C at night (Table 1), this phenomenon will show apparent improvement and also greatly slow down the loss of hot air when the plants in the warming unit construct a relatively closed space with the surrounding transparent plastic walls (Fig. 7). Considering the amplitude of temperature increase at the different sites, point C was selected as the temperature measurement point of each main plot.

**Table 1:** Average increments of different positions in a plot at daytime and nighttime

Period	A	B	C	D	E
Daytime increment/ °C	1.04	0.56	0.98	0.65	0.82
Nighttime increment/ °C	1.73	0.98	1.26	1.04	1.37

The temperature data in the table are the difference between the mean temperature of warming plot and normal temperature in the corresponding warming period (daytime, 7:00–19:00; nighttime, 19:00–7:00 the next day) at different sites. A–E represents different position points of the plot, and their relative positions are shown in Fig. 6

### Verifying the warming effect of the HBWF

#### Temperature increment

The hot-blast warming facility had a conspicuous warming effect in the field that could raise the air temperature in the rice canopy by 1.0–2.1 °C during the whole growth period (Table 2). The diurnal average increment and average increment in daytime and nighttime in 2015 were all significantly higher than those in 2016. The average increment at nighttime, daytime and the diurnal of the NW, DW, and AW treatments were 2.09 °C, 1.17 °C and 1.85 °C in 2015, respectively, and 1.32 °C, 0.97 °C and 1.12 °C in 2016, respectively. An asymmetry of temperature increment between the daytime and nighttime in the future could be observed from these results; furthermore, the asymmetry was also reflected in the average increment at daytime and nighttime of the AW treatment. Overall, the HBWF achieved the expected purpose in simulating the changing trend of global warming in the future.

**Table 2:** Average increments of rice canopy temperature during the whole growing duration under three warming treatments

Treatment	All-day warming		Day warming		Night warming	
	2015	2016	2015	2016	2015	2016
Diurnal average increment	1.85	1.12	–	–	–	–
Average increment at daytime	1.54	0.68	1.17	0.97	–	–
Average increment at nighttime	2.17	1.53	–	–	2.09	1.32

The temperature data in the table are the difference of mean temperature between different warming treatments (Daytime: 7:00–19:00, Nighttime: 19:00–7:00 the next day, All day: 7:00–7:00 the next day) and the normal temperature

#### Variation characteristics of temperature

**Fig. 8:**
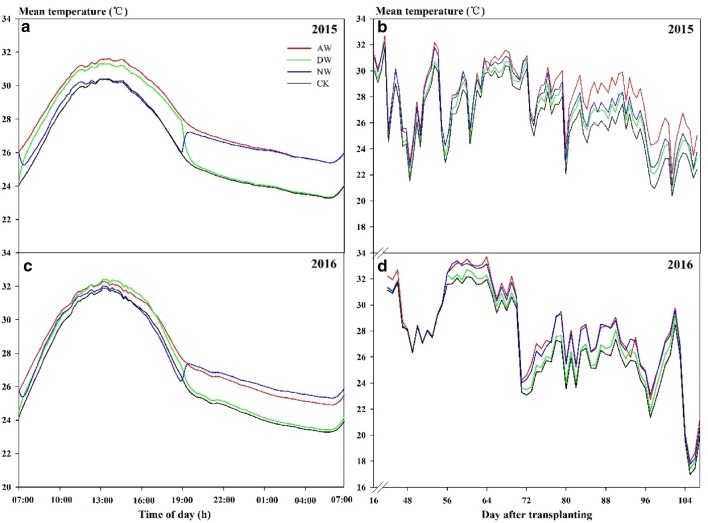
Trends of diurnal temperature variation and diurnal mean temperature of rice canopy during the whole growth duration under different warming treatments in 2015 **a**, **b** and 2016 **c**, **d**. AW, DW, NW, and CK represent all-day warming, day warming, night warming and ambient control, respectively

With the HBWF, the diurnal variation dynamics of the average rice canopy temperature under three different temperature warming treatments were nearly consistent with the natural control according to the daily variation in all of the stages over 2 years dynamic curve (Fig. 8a, c). In both years, greater warming was achieved from 19:00 to 07:00 (of the next day) during the all-day and nighttime warming treatments, and the fluctuation of the temperature increment was significantly reduced in 2016 compared with 2015.

Data regarding the daily average temperature of different warming treatments during the whole growing period in 2015 and 2016 are presented in Fig. 8b, d. The data revealed that the average atmospheric temperature increased significantly under the three warming treatments compared with the control, while there was a significant variation of temperature increment among the treatments (AW > NW > DW). In 2016, there was a period of high temperature with the maximum diurnal mean temperature reaching almost 34 °C between 55 days and 65 days after transplanting, and this period coincided with the flowering period of rice. The actual daily maximum temperature exceeded the heat stress threshold for this stage. In contrast, the daily average temperature of the flowering period was lower while the temperature fluctuated greatly in 2015.

#### Relative humidity

There were significant effects caused by the warming treatments on the relative humidity of the rice canopy in the warming plot compared to the control treatment during the whole growth duration in both years (Table 3). The data demonstrated that the relative humidity of the rice canopy was significantly reduced by each warming treatment in both years. The relative humidity of all treatments decreased by 5.11%, 2.51%, and 9.30% in the NW, DW and AW treatments in 2015, respectively, while a mild reduction of 3.49%, 2.05%, and 4.18% occurred in the NW, DW, and AW treatments in 2016, respectively. The absolute value of the relative humidity change between the three treatments in 2016 was 3.5%, while in 2015 it was recorded 8%, which was significantly higher than the 2016 observed value. Although the relative humidity of the rice canopy was significantly different among the different treatments, the absolute value of the relative humidity revealed that the fluctuation range of this value among the four temperature treatments was 79–87%. This indicated that the relative humidity value of the rice canopy was larger in general and its fluctuation range was very limited during the whole growth duration in the rice ecosystem.

**Table 3:** Effects of three warming treatments on the relative humidity of rice canopy during the whole growing duration in 2015–2016

Treatment	Year		Diff (%)	
	2015	2016	2015	2016
CK	87.50 ± 0.29 a	84.06 ± 1.11 a	–	–
NW	83.03 ± 1.17 b	81.13 ± 0.57 b	− 5.11	− 3.49
DW	85.30 ± 0.61 ab	82.34 ± 1.25 ab	− 2.51	− 2.05
AW	79.36 ± 1.12 c	80.55 ± 0.74 b	− 9.30	− 4.18

AW, DW, NW, and CK represent all-day warming, day warming, night warming, and ambient control. Means followed by different letters are significantly different at a probability level of 0.05 according to the Least Significant Difference (LSD) test

#### Grain yield

**Fig. 9:**
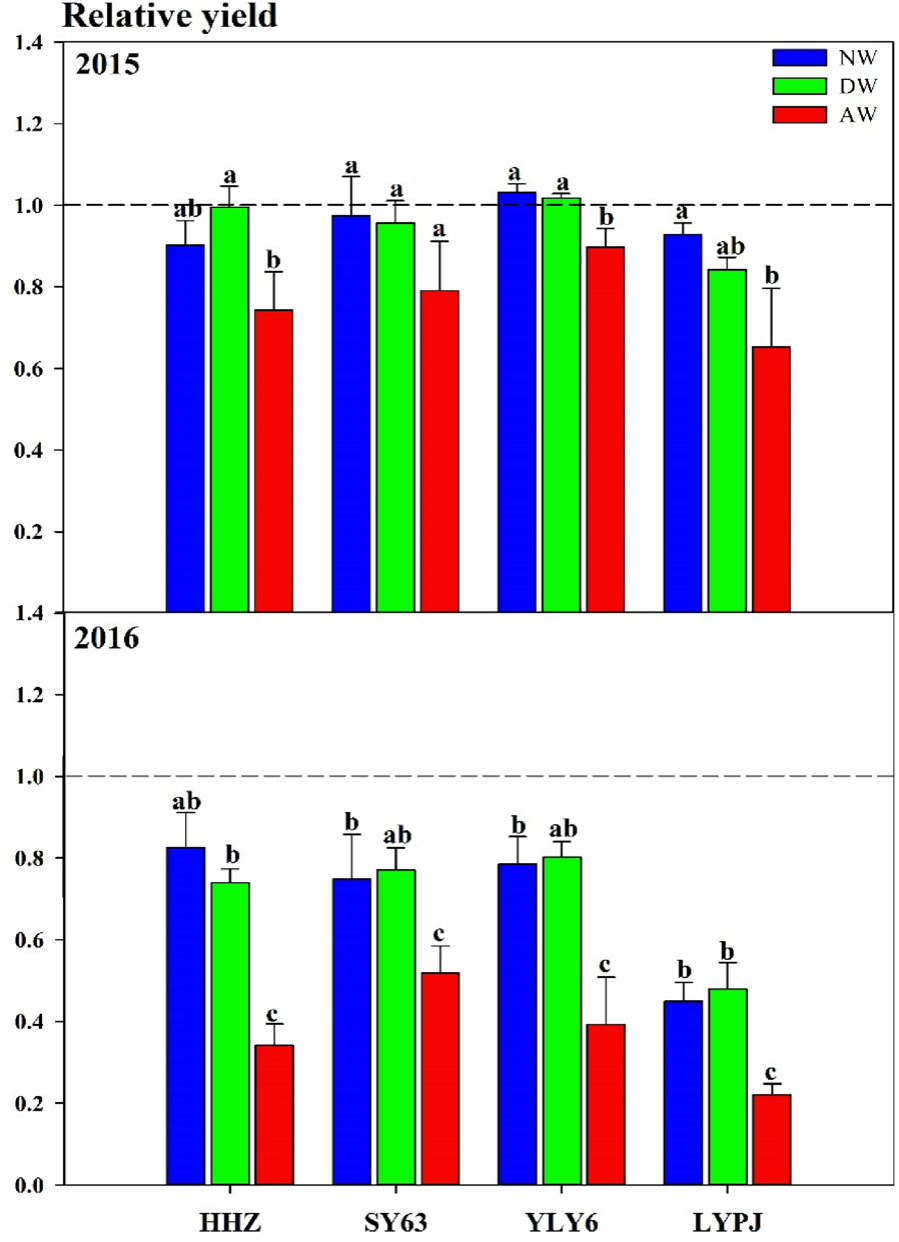
Changes in rice relative yield under three warming treatments in 2015 and 2016. The abbreviations HHZ, SY63, YLY6, and LYPJ stand for varieties of Huanghuazhan, Shanyou63, Yangliangyou6, and Liangyoupeijiu, respectively. AW, DW and NW represent all-day warming, day warming and night warming, respectively. Means followed by different letters are significantly different at a probability level of 0.05 according to the Least Significant Difference (LSD) test and error bars above denote standard error (SE) of the replications (n = 4)

Taking the rice yield under CK as the benchmark, the data of the relative yield intuitively showed the changes in rice yield under warming treatments. Our trials demonstrate that a temperature variation of approximately 2 °C can lead to significant yield losses (Fig. 9). Among the tested elevated temperature treatments, the adverse effect of AW was the largest, while the effects of DW and NW on rice yield were consistent. In 2015, the rice yield was reduced by 26.1%, 19.0%, 10.5%, and 35.2% by AW treatment for HHZ, SY63, YLY6, and LYPJ, respectively. During the second year of the AW trial, the yield decreased by 65.9%, 42.8%, 61.0%, and 77.9% for HHZ, SY63, YLY6, and LYPJ, respectively. Compared with CK, the effects of DW and NW on rice yield reduction were not significant in 2015, however, in the next year DW decreased the yield by 26.3%, 25.7%, 20.1%, and 52.1% for HHZ, SY63, YLY6, and LYPJ, respectively, and the NW rice yield was reduced up to 17.8%, 28.4%, 22.0%, and 54.8% for HHZ, SY63, YLY6, and LYPJ, respectively.

Considering the cultivar variation, the data in Fig. 9 revealed that the grain yield reduction in SY63 was the lowest of all four tested varieties in the NW, DW, and AW treatments in both years; additionally, the reduction in LYPJ was the greatest among the elevated-temperature treatments of all varieties. Moreover, Fig. 9 clearly shows that the adverse impact of the heating on rice in 2016 was significantly greater than that in 2015.

#### Grain yield components

**Fig. 10:**
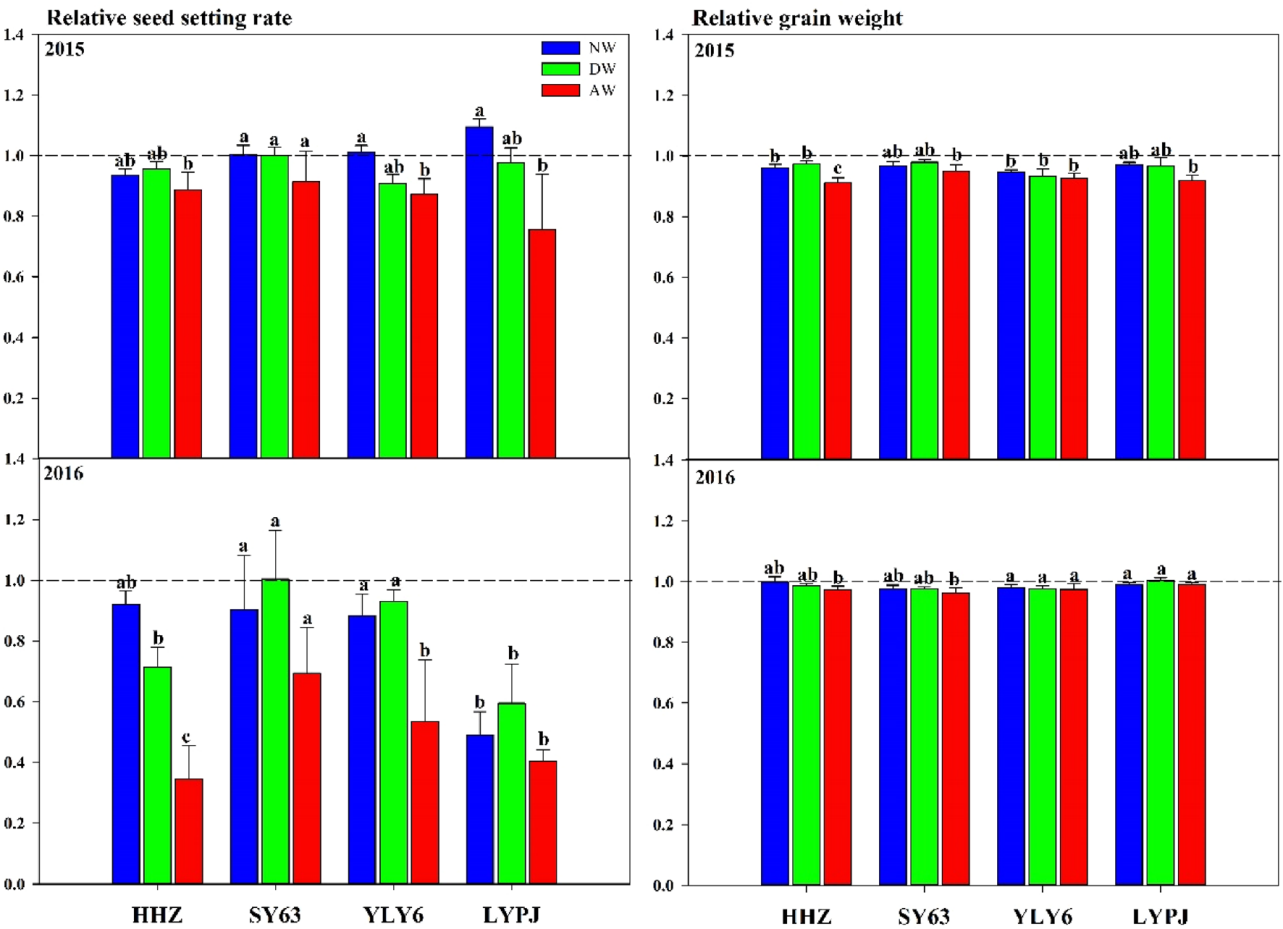
Effects of three warming treatments on relative seed setting rate and relative grain weight in 2015 and 2016. The abbreviations HHZ, SY63, YLY6, and LYPJ stand for varieties of Huanghuazhan, Shanyou63, Yangliangyou6, and Liangyoupeijiu, respectively. AW, DW and NW represent all-day warming, day warming and night warming, respectively. Means followed by different letters are significantly different at a probability level of 0.05 according to the Least Significant Difference (LSD) test and error bars above denote standard error (SE) of the replications (n = 4)

By analyzing the yield components of the four varieties during 2015 and 2016, the results showed that the decrease in rice yield was mainly caused by a decrease in the seed setting rate, followed by a decrease in the 1000-grain weight (Fig. 10). The data documented that the effect of the AW treatment on the seed setting rate was the greatest. In 2015 and 2016, the seed setting rates of AW decreased by 8.6–24.4% and 30.6–65.6%, respectively (Fig. 10a, c). The mean value of the 1000-grain weight of the four tested cultivars was significantly decreased by 1.7% in 2016, which was significantly lower than that in 2015 (5%). No distinct and consistent variation in the effective panicles per unit area and the number of spikelets per panicle was observed in the four varieties under AW, DW, and NW when compared with the ambient treatment in both years (data not shown).

### Discussion

To study the potential effects of global warming on different terrestrial ecosystems (such as grassland and crops), previous studies manipulated temperature in the field with a variety of warming facilities, including greenhouses [17], open-top chambers (OTCs) [20], soil heating pipes [29], infrared reflectors [30] and infrared heaters (Free Air Temperature Increase, FATI) [31, 32]. However, to simulate the global warming mechanism in natural conditions and obtain asymmetric warming in the daytime and nighttime, only the FATI system can better satisfy the requirements at present. The FATI device adopts the traditional method of infrared radiation crop foliage warming [26] and can’t simulate air convection heating [27]. The HBWF provides an additional option based on OTCs that raises temperature through heating air directly with non-destructive (Fig. 7). The facility completely avoids the solar radiation shading effects by infrared heaters and makes up for the deficiency observed in other methods to simulate air convection heating while not altering the spectral characteristics.

#### Temperature increase

Our data show that the new-type of warming facility had a conspicuous warming effect in the field that could raise the air temperature in the rice canopy by 1.0–2.1 °C (Table 1). Similar temperature enhancements were achieved in some previous studies [28, 33]. According to inference by the IPCC [1], the temperature increment is appropriate in the HBWF and is in line with the predicted range of atmospheric temperature increases in China in the next few decades. In a warming unit, the vertical temperature gradient was studied and the results indicated that the temperature decreases with the height increasing. From 55 cm to 75 cm, there’s about a 2 °C drop in temperature. Although we formed a relatively closed space through the rice plants and transparent plastic walls (Fig. 7), the hot air flows from the lower part of the rice to the canopy, so there are a certain lag and heat dissipation during the facility operation. Besides, it could show the characteristics of asymmetry about global warming in the daytime and nighttime (Table 2). In the context of global warming, the ascending range of the highest temperature in the daytime is much lower than that of the lowest temperature at night [34–37], and there are enormous differences in the impact of elevated temperatures on rice between daytime and nighttime [10]. So this feature is undoubtedly important for a warming trial in the field. During the daytime and nighttime, the facility could raise the air temperature in the rice canopy by 0.6–1.5 °C and 1.3–2.2 °C, respectively (Table 2). It satisfied the requirement of field warming experiments well. Under the HBWF, there was significant variation of temperature increment in diurnal mean temperature of rice canopy among the treatments (AW > NW > DW) (Fig. 8b, d) and the trends of diurnal temperature variation dynamics under warming treatments were nearly consistent with the natural control (Fig. 8a, c). Such warming circumstances can better reflect the actual changes in rice plants under future climate change. However, the warming effect of the facility will be affected by the foundational atmospheric temperature between different years. The research results show that the temperature increment of each warming treatment in 2015 was higher than that in 2016 (Table 2). According to the temperature record under 2 years of natural conditions, the average atmospheric temperature in 2016 was higher than that in 2015, and the number of days with high temperature (≥ 35 °C) in 2016 was 37 days (data not shown), which was much higher than that in 2015. This may be the causes for the difference in the warming effect of the facility in 2 years. If the facility is to continue to be used in field trials with a steady warming effect in elevated temperature, the above changes may warrant attention and improvement (e.g., by increasing the power of the heater).

#### Relative humidity

Among common environmental factors, not only temperature but also humidity can affect the growth and development of rice [38]. One problem that had been identified in the heating experiment with infrared heaters (FATI) is that the vapor pressure gradients (VPGs) from inside the leaves to the air outside would not be the same as would be expected [24]. Therefore, we investigated the changes in relative humidity over the whole growth period of each main plot in this trial. The findings regarding relative air humidity imply that the absolute value of relative humidity fluctuated very little between the four temperature treatments, despite the relative humidity of the rice canopy being significantly different among the different treatments (Table 3). Moreover, we calculated the average relative humidity of natural air during the rice-growing season of 2015–2016. The relative humidity were 75.2% and 72.0% in 2015 and 2016, respectively. Meanwhile, humidity varied in the range of 79–87% in 2015 and 80–84% in 2016, which were a higher humidity level and were all bigger than the average relative humidity of natural air. It’s demonstrated that although the vapor pressure deficit in the air will vary, the changes are very limited in the paddy field when the air is heated directly by simulating convective heating.

For this problem with VPGs, Kimball [24] proposed that irrigation can be used to compensate for the water lost in the ecosystem as a first-order correction. In this assessing study, there was adequate moisture present in the field of the irrigated rice system because the paddy field was flooded in a long term. As a consequence, the HBWF has a limited impact on the relative humidity of the heated plot under the paddy field. Until now, this facility was only used in a rice planting system, and land surface temperature and soil moisture were not investigated in this paper because we maintained flood irrigation management during the growth period. If we want to study the response of wheat and other dryland crops to elevated temperatures, we would need to consider the influence of temperature enhancement on soil temperature, air humidity, soil moisture content and so on just like Wan et al. [23].

#### Advantages of the facility and precautions for using

The warming effect of an open-field warming facility that composed of infrared heaters usually decreased with increasing wind speed [23], therefore these facilities usually need to deploy a proportional-integrative-derivative (PID) control system and infrared thermometers (IRTs) to prevent the wind from weakening the output of infrared heaters and increase temperature stability [24, 26]. In the HBWF, the role of PID and IRTs are replaced by transparent plastic walls. Four transparent plastic walls forming a wind break were installed as a thermal insulation device around each plot and formed a relatively closed space for the rice plants (Fig. 7). This heating method with active and passive characteristics not only simplified the equipment layout but also isolated adjacent areas so they did not affect each other. This also saved space and improved the land utilization effectiveness of the field block. Nevertheless, in early stages of the growth, the plants close to the walls were also taller, indicative of less mechanical disturbance by wind. These effects were taken into account to ensure the accuracy of the trials when sampling. Additionally, the same position perpendicular to the direction of the pipe was selected for each destructive sampling, and the original position was filled by transplanting other marked rice plants after sampling to ensure the tightness of the group space and avoid the border effect. After the HBWF was started, access into the warming unit should be reduced as far as possible to avoid the interference of human factors.

Currently, a FATI system needed to deploy six infrared heaters with a rated power of 1 kW to form an effective warming unit with an area of 7 m^2^ in the paddy field [26]. It consumed approximately 0.85 kW per square meter. This meant that if we wanted to use the FATI system to complete a warming test, we would have a high cost of operating. In contrast, the HBWF consumed only approximately 0.165 kW per square meter and reduced operating costs by 80% because it doesn’t operate in free air. Furthermore, installation convenience, facility security, and thermal insulation of the new equipment were greatly improved compared to those of predecessors [28]. In the HBWF, the heater, blower, and rainproof cover were all connected and fixed by screws, and the cables of the equipment were arranged into PVC pipes in advance. Compared with the FATI [26], the HBWF was easy to install and remove due to the cables and components were greatly reduced. Nevertheless, due to the facility running in a field environment, its line laying was installed by professional electricians based on the field design, and its daily maintenance and management were also handled by the professional and technical personnel. Also, the facility should be turned off to improve safety when sampling, although a variety of electrical leakage protection measures (Figs. 2b, d, 3e, h) were taken during operation. However, it is undeniable that when the new warming facility is used to conduct a warming experiment during the whole growing period of crops, higher electricity costs will be generated (for example, the total energy consumption in our trial is 70,848 kW h in 90 days). Therefore, it would be more feasible to consider multi-party cooperation or to select a specific growth period of crops for a warming experiment for laboratories with insufficient funds. Overall, the hot-blast warming facility was suitable for the fixed-point test with several temperature treatments, combinations, and plot arrangements and can be used for many years. The HBWF provides an extra equipment option for use in elevated temperature research in the future.

#### Grain yield and yield components

Temperature is an important environmental factor in controlling panicle growth and ripening [39]. The rise of the diurnal mean maximum and minimum temperatures under global warming will have adverse effects on the formation of rice yields [40, 41]. Here, our trials demonstrate that a temperature variation of approximately 2 °C can lead to significant yield losses (Fig. 9). The experimental results were consistent with the reduction trend predicted by previous models [9]. During both years, the maximum decrease in grain yield was recorded under AW treatments. In 2015 and 2016, the yield in the AW treatment was reduced by 10–35% and 42–79%, respectively. The conspicuous reduction of AW was ascribed to the uninterrupted elevated temperatures for the diel (24 h), which formed a superposition effect of the DW and NW treatments and resulted in a comparatively higher cumulative temperature at the end of the growth period. Such a reduction in yield with an all-day temperature increase had been already reported in various investigations [28], but Dong et al. [33] reported that the rice yield was decreased only by 0.9% under AW, which was far less than the decrease of yield under DW and NW. These differences might be attributed to the interannual differences in the natural temperature of the atmosphere, which led to the stronger or weaker responses of rice to the elevated temperatures. Our results of both years confirmed this inference. Meanwhile, ecotypes of the tested rice and other environmental factors such as radiation, relative humidity, and rainfall have impacted the growth of rice in a season, leading to the differences in results obtained from different studies. The reduction of yield in four cultivars in DW and NW was less than in AW, but the adverse impacts of these cultivars were consistent. The effect of DW and NW on the rice yield during 2015 was not significant, while DW and NW significantly decreased the rice yield of four cultivars in 2016. Peng et al. [10] reported that grain yield declined by 10% for each 1 °C increase in the nighttime temperature, while daytime temperature had an inconspicuous influence on the rice yield. In 2016, the yield was reduced by 18–55% when the nighttime temperature increased by 2 °C, which was approximately similar to Peng’s result. However, the DW treatment diminished the grain yield by 20–52% in 2016, which was different from the results of Peng et al. [10]. We speculated that this difference was mainly ascribed to the natural temperature level in 2016 is higher than that of the past years. According to the temperature data under 2 years of natural conditions, the number of days with high temperature (≥ 35 °C) was 37 days and there was a period of high temperature during the 55–65 days after transplanting in 2016 (Fig. 8d), which coincided with the flowering period of rice. This meant that the frequency of suffering high-temperature stress increased for rice plants in the DW treatment and aggravated the high-temperature stress response. The responses of the four indica ecotypes to warming treatments were also significantly different. Our results demonstrated that the performance of LYPJ was the worst; simultaneously, SY63 and YLY6 showed more tolerance than the other two genotypes under elevated temperatures (Fig. 9). In the same ecotype, the two-line hybrid rice LYPJ possessed relatively more sensitive to temperature increments than other cultivars. Furthermore, Shah et al. [28] revealed that japonica ecotypes were much more sensitive to heat stress than indica ecotypes concerning yield. Thus, an effective way to reduce the detrimental effects of high temperatures is by selecting and breeding high temperature-resistant varieties from different ecological types and determining the genetic differences of the varieties with different temperature sensitivity in response to a warming climate.

Rice grain yield is determined by four parameters: number of panicles per plant, number of spikelets per panicle, grain filling rate and total grain weight [42]. With global warming, the increase in the maximum daily temperature will significantly increase the frequency of suffering from high-temperature stress for rice during the day [43], which is not conducive to the reproductive growth of rice [44, 45] and seriously affects the seed setting rate and the 1000-grain weight of rice [46]. By analyzing the yield composition factors of the four varieties during 2015 and 2016, it can be found that the decrease in rice yield was mainly caused by a decrease in the seed setting rate, followed by a decrease in the 1000-grain weight (Fig. 10). In 2016, an approximately 60% reduction in the seed setting rate was observed in LYPJ and HHZ under AW treatment. In contrast, although the 1000-grain weight of rice was significantly reduced by 5% under the different warming treatments, especially the AW treatment, it did not contribute much to the grain yield reduction. Hubei Province, as the main producing area of middle-season rice in the middle and lower reaches of the Yangtze River, is prone to encounter high-temperature stress during the fertile flowering period under warming weather, which will inhibit pollen activity, anther cracking, and stigma pollen germination, leading to a high sterility of spikelets and a reduced seed setting rate [44, 47]. Under the trend that the average atmospheric temperature will continue to rise in the future with climate warming, it can be predicted that rice production in the middle and lower reaches of the Yangtze River will face a severe test based on the results of this study. In this context, it is of great significance to design stable and reliable field warming facilities and observe an accurate simulation of future climate change. The area of temperature increase in the HBWF is sufficiently large to meet the needs of multiple samples, and an in-depth exploration can be conducted on the internal mechanisms between rice yield composition factors and the temperature increases in the future research. Not only that, it’s convenient to explore the role of field cultivation means such as fertilization and irrigation in alleviating the temperature increase effects through the new warming facility.

## Conclusions

An additional field warming facility option named as hot-blast warming facility (HBWF), which comprising heaters, blowers, wind breaks, and a control board was developed. The modular design of the facility facilitated the installation and disassembly of different devices, which also greatly improves the safety of the equipment. The farmland hot-blast warming facility could raise the temperature to 1–2 °C in the rice canopy without changing the diurnal variation characteristics and formed form an effective warming unit with an abundant area. The warming units are independent of each other, which enables carrying out multiple elevated temperature treatments for different crops in the limited experimental land at the same time. Furthermore, the HBWF had little effect on relative humidity in paddy field and could simulate convection heating by directly heating air. The power consumption per square meter in the warming plot was reduced due to the adoption of heat preservation equipment. In the validation trial, the facility stably provided uniformly controlled warming plots, and it provided reproducible results under field conditions. Among all treatments, the AW caused the most adverse effects, and the day and night temperature augmentation caused the same degree of adverse effects. The primary cause for the significant decrease in rice yield in 2 years was a decrease in the seed setting rate, followed by a decrease in the 1000-grain weight. In general, the hot-blast warming facility developed in this experiment and the resultant data from rice planted in middle-season are of great reference value to predict the specific changes of rice under the future climate warming and to propose feasible countermeasures accordingly.

## Methods

### Site description

Field warming experiments were conducted during July–October in 2015–2016 using a hot-blast facility at Huazhong Agricultural University, Wuhan, Hubei Province, China (30° 50′ N latitude, 114° 33′ E longitude). The climate conditions can be classified as a northern subtropics monsoon with a mean annual precipitation of 1300 mm, a mean annual temperature of 17.1 °C, and an annual daylight period of 1800 h. The relevant soil properties were as follows: total nitrogen 1.20 g kg^−1^, available phosphorus 10.47 mg kg^−1^, extractable potassium 125.92 mg kg^−1^, soil organic carbon 16.62 g kg^−1^, and pH 6.32.

### Crop establishment

Four indica rice (*Oryza sativa L.*) cultivars having different sensitivity to high temperature, Huanghuazhan (HHZ), Shanyou63 (SY63), Yangliangyou6 (YLY6) and Liangyoupeijiu (LYPJ), were tested consecutively for the 2-year warming experiment. Sterilized seed of all four cultivars was germinated and then sown on 11th May and 17th May for the nursery rearing in 2015 and 2016, respectively. One month after sowing, three seedlings per hill were transplanted manually with a hill spacing of 16.7 cm × 20.0 cm in mid-June. The plants were cultivated in a paddy for the whole rice growth duration. The rates of fertilizers in each plot were 180 kg N ha^−1^, 40 kg P (P_2_O_5_) ha^−1^, and 100 kg K (KCl) ha^−1^. All of the P, half of the K and 40% of N were applied as a basal dressing 1 day before transplanting, while the residual nitrogen was equally split at the mid-tillering stage and the panicle initiation stages, and the other 50% of the potassium was top-dressed during panicle initiation. All the weeds were manually removed whenever they were found, and various pesticide sprays were used for pest and disease control.

### Experimental design

A warming experiment was conducted in the HBWF according to split plot arrangements with four replicates. Elevated temperature treatments were allocated to the main plots having an area of 25 m^2^ (5 m × 5 m) (Fig. 5). Each main plot was divided into four subplots equally and four cultivars were assigned randomly to the subplots. The treatments were all-day warming (AW: warmed from 0: 00 to 24: 00), day warming (DW: warmed from 7: 00 to 19: 00), night warming (NW: warmed from 19: 00 to 7: 00 of the next day) and ambient control (CK). The warming treatments were deployed after transplanting, from 25/06 to 29/09 in 2015 and from 29/07 to 08/10 in 2016. The start time of the HBWF was delayed because of continual rainfall after transplanting in 2016.

### Grain yield and its components

At physiological maturity, 50 hills from each cultivar in the center of each replicate plot were harvested, and the grain weight was adjusted to 14% moisture content in 2015 and 2016. Besides, plants of 12 hills from each plot were sampled to determine the yield components. The number of panicles m^−2^ and spikelets per panicle were calculated along with the percent seed-set and the 1000-grain weight.

### Data collection and analysis

Canopy air temperature data derived from HOBO Micro Station data logger were also averaged to obtain daytime, nighttime, and diel means, and then the increment was obtained by comparing the different warming treatments with the corresponding period of CK. The relative humidity data of the canopy during the whole growth period in each main plot were averaged, and then the variance analysis was carried out according to the different treatments. Analysis of variance was performed with Statistix 9.0 using the least significant difference (LSD) test at a 0.05 probability level. All figures were constructed using Sigma Plot 13.0.

## Data Availability

All data generated or analyzed during this study are included in this published article (and its additional file).
